# ST-Segment Elevation: An Unexpected Culprit

**DOI:** 10.3390/jcdd10090374

**Published:** 2023-09-01

**Authors:** David Sá Couto, André Alexandre, Ricardo Costa, Andreia Campinas, Mariana Santos, Diana Ribeiro, Severo Torres, André Luz

**Affiliations:** 1 Cardiology Department, Centro Hospitalar Universitário de Santo António, Largo do Prof. Abel Salazar, 4099-001 Porto, Portugal; u13404@chporto.min-saude.pt (A.A.); ricardofcosta.cardiologia@chporto.min-saude.pt (R.C.); andreiacampinas.cardiologia@chporto.min-saude.pt (A.C.); u14204@chporto.min-saude.pt (M.S.);; 2 ICBAS School of Medicine and Biomedical Sciences, University of Porto, Rua Jorge Viterbo Ferreira 228, 4050-313 Porto, Portugal; 3 Cardiovascular Research Group at Unidade Muldisciplinar de Investigação Biomédica (UMIB), ICBAS School of Medicine and Biomedical Sciences, University of Porto, Rua Jorge Viterbo Ferreira 228, 4050-313 Porto, Portugal

**Keywords:** ST-segment, acute pulmonary embolism, catheter-directed therapy, intervention cardiology, thrombolysis, aspiration thrombectomy

## Abstract

The clinical presentation of pulmonary embolism (PE) and acute coronary syndrome can be similar. We report a case of a patient presenting with antero-septal ST-segment elevation after cardiac arrest, found to have acute-PE-mimicking ST-segment elevation myocardial infarction (STEMI), treated with aspiration thrombectomy and catheter-directed thrombolysis (CDT). A 78-year-old man was admitted with dyspnea, chest pain and tachycardia. During evaluation, cardiac arrest in pulseless electrical activity was documented. Advanced life support was started immediately. ECG post-ROSC revealed ST-segment elevation in V1–V4 and aVR. Echocardiography showed normal left ventricular function but right ventricular (RV) dilation and severe dysfunction. The patient was in shock and was promptly referred to cardiac catheterization that excluded significant CAD. Due to the discordant ECG and echocardiogram findings, acute PE was suspected, and immediate invasive pulmonary angiography revealed bilateral massive pulmonary embolism. Successful aspiration thrombectomy was performed followed by local alteplase infusion. At the end of the procedure, mPAP was reduced and blood pressure normalized allowing withdrawal of vasopressor support. Twenty-four-hour echocardiographic reassessment showed normal-sized cardiac chambers with preserved biventricular systolic function. Bedside echocardiography in patients with ST-segment elevation post-ROSC is instrumental in raising the suspicion of acute PE. In the absence of a culprit coronary lesion, prompt pulmonary angiography should be considered if immediately feasible. In these cases, CDT and aspiration in high-risk acute PE seem safe and effective in relieving obstructive shock and restoring hemodynamics.

## 1. Introduction

Acute pulmonary embolism (PE) is associated with significant morbidity and mortality, having multiple possible clinical presentations, most of them unspecific [1,2]. Acute onset dyspnea and chest pain are common presenting symptoms, which largely overlap with acute coronary syndrome manifestations [1,3].

In most patients with acute onset chest pain or dyspnea, the electrocardiogram (ECG) is the first diagnostic test to be performed. ST-segment elevation is usually associated with myocardial infarction (MI) and, in the right clinical setting, should prompt immediate admission to the cardiac catheterization laboratory (CCL) and revascularization [4]. Nevertheless, there are alternative causes of ST-segment elevation, such as pericarditis/myocarditis, Takotsubo cardiomyopathy, channelopathies, benign early repolarization, electrolyte abnormalities and acute PE, among others [5].

In large central PE, acute increases in pulmonary arterial pressure and thus right ventricle (RV) afterload can result in RV dilation and dysfunction precipitating obstructive shock and even cardiac arrest. This ventricular straining might be associated with ischemia and myocardial injury which can rarely manifest itself as ST-segment elevation [6]. Alternatively, other theories have been proposed, stating that oxygen delivery/consumption mismatch, during right ventricular strain, might be implicated in epicardial or microvascular coronary artery vasospasm, inducing ischemia [7]. In the setting of hemodynamic instability, point-of-care echocardiography can be a helpful strategy in diagnostic orientation [3]. However, RV dysfunction can also be present in myocardial infarction (MI) with RV involvement.

In the face of ST-segment elevation and acute chest pain, dyspnea or hemodynamic instability, the decision is often to proceed to coronary angiography. When no epicardial coronary artery disease (CAD) is found, alternative causes for ST-segment elevation must be considered, one of them being acute PE [4].

Catheter-directed treatment (CDT) of PE has been growing as a useful technique in patients with contraindications to systemic thrombolysis (STL) or standard treatment failure. Trials have been published reporting on the safety and efficacy of aspiration thrombectomy and catheter-directed thrombolysis in the treatment of intermediate-high-risk PE [8,9,10,11,12,13]. Robust evidence of these strategies for high-risk cases is still lacking. Multiple devices have been developed for CDT, the Penumbra^®^ Indigo^®^ (Alameda, California, United States of America) system being one of the validated options [11].

In this article, we report a case of a patient presenting with antero-septal ST-segment elevation after cardiac arrest. A diagnosis of acute PE, mimicking ST-segment elevation MI (STEMI), was made using invasive pulmonary angiography. He was treated with catheter-directed thrombolysis and aspiration thrombectomy using the Penumbra^®^ Indigo^®^ thrombus aspiration system.

## 2. Detailed Case Description

A 78-year-old man, with a history of dyslipidemia and Herpes Zoster ophthalmicus (complicated with meningitis 4 months prior), medicated with prednisolone, pregabalin, calcium carbonate and cotrimoxazole, presented to the emergency department. He reported worsening exertional dyspnea for the past 4 days and mild chest pain localized to the left hemithorax. At admission, he was febrile (tympanic temperature of 38 °C), mildly tachycardic (115 beats per minute), blood pressure (BP) was 113/79 mmHg, and peripheral oxygen saturation (SpO_2_) was 93%. Physical examination was unremarkable. Arterial blood gas (ABG) analysis at room air showed pH 7.48; pCO_2_ 32 mmHg; pO_2_ 70 mmHg; and lactate 2.5 mmol/L.

**Figure 1:**
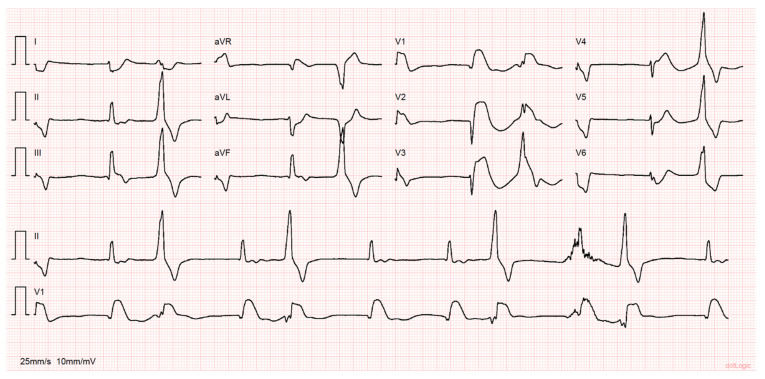
Twelve-lead electrocardiogram preformed after return of spontaneous circulation. Note the irregular rhythm with a few monomorphic premature ventricular complexes and ST-segment elevation in V1, V2, V3 and aVR, with ST-segment depression in DI, aVL and V6.

While under evaluation, the patient went into cardiac arrest in pulseless electrical activity. Advanced life support was started immediately with return of spontaneous circulation (ROSC) after 25 min. Prior to ROSC, ventricular fibrillation was noted, and two shocks were delivered. During the resuscitation maneuvers, he was intubated and mechanically ventilated. The ECG post-ROSC revealed an irregular rhythm with a few monomorphic premature ventricular complexes, ST-segment elevation in V1–V4 and aVR and ST-segment depression in DI, aVL and V6 (Figure 1). Point-of-care echocardiography demonstrated mild aortic regurgitation, an absence of signs suggesting aortic dissection or pericardial effusion, preserved left ventricular systolic function, severe hypokinesis of the interventricular septum and right ventricle (RV) dilation with severe systolic dysfunction.

**Figure 2:**
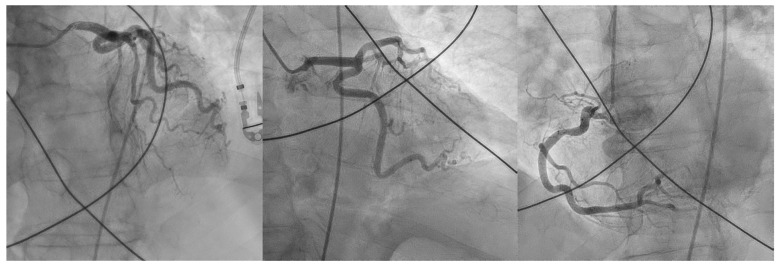
Coronary angiography showing absence of clinically significant epicardial coronary artery disease: left anterior descending (left picture) has a 50% stenosis, circumflex (middle picture), and right coronary artery (right picture) shows only mild irregularities.

The patient was in shock and in severe respiratory failure. Considering an eventual MI, despite the discordant echocardiographic assessment, he was emergently transferred to the CCL due to the exuberant ST-segment elevation post-ROSC. Coronary angiography showed an intermediate stenosis (50%) of the left anterior descending coronary artery and an absence of epicardial disease in the other vessels (Figure 2, Videos S1, S2 and S3).

**Figure 3:**
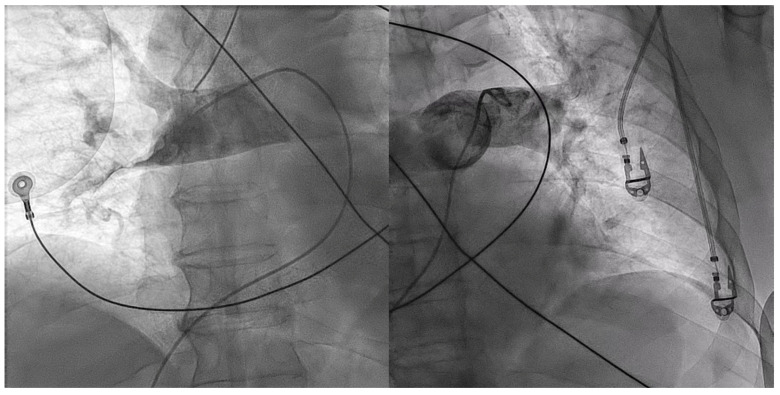
Pulmonary angiography showing bilateral massive pulmonary embolism with complete proximal occlusion of the right pulmonary artery (left picture) and left lobar branch thrombotic occlusion (right picture).

During catheterization, hemodynamic status remained poor with the need for vasopressor support. Due to the high clinical suspicion of acute PE, immediate procedural conversion to right heart catheterization was performed, revealing a mean pulmonary artery pressure (mPAP) of 45 mmHg. Subsequent pulmonary angiography showed bilateral massive pulmonary embolism with complete proximal occlusion of the right pulmonary artery (Figure 3, Videos S4 and S5).

**Figure 4:**
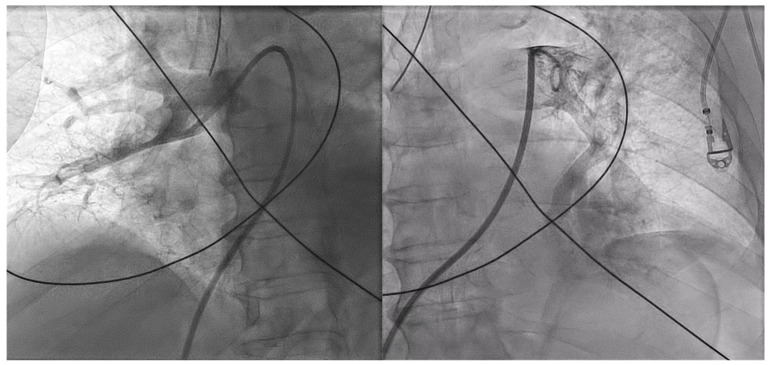
Pulmonary angiography after aspiration thrombectomy and bilateral alteplase bolus. There was perfusion improvement despite the remaining high thrombotic burden and significant distal embolism.

Percutaneous thrombectomy was attempted with Penumbra^®^ Indigo^®^ System 8F. Multiple passages with a separator wire, 10 mg of alteplase injected in each pulmonary artery and 7000 UI of systemic unfractioned heparin (UFH) were successful in recanalizing the major vessels (Videos S6 and S7). Nevertheless, there was still a significant remaining thrombotic burden and distal embolism (Figure 4, Videos S8 and S9).

**Figure 5:**
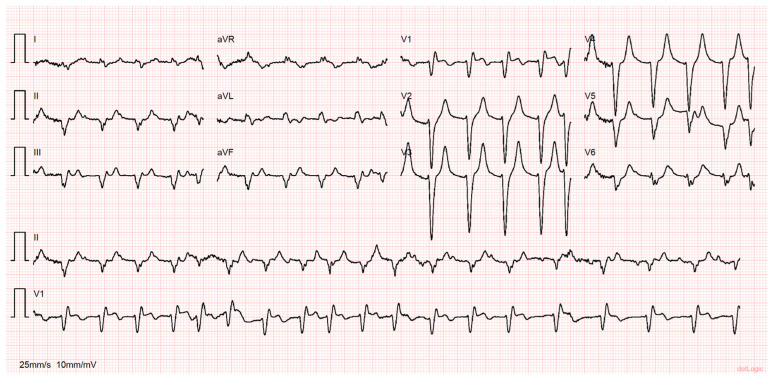
Postprocedural ECG showing resolution of the expressive ST-segment elevation seen at admission. The patient was in atrial fibrillation and showed significant intraventricular conduction abnormalities, left axis deviation and persisting millimetric ST-segment elevation in V1.

At the end of the procedure, mPAP was 35 mmHg and BP normalized without the need for further vasopressor support. Postprocedural ECG showed resolution of the previously described ST-segment elevation (Figure 5).

**Figure 6:**
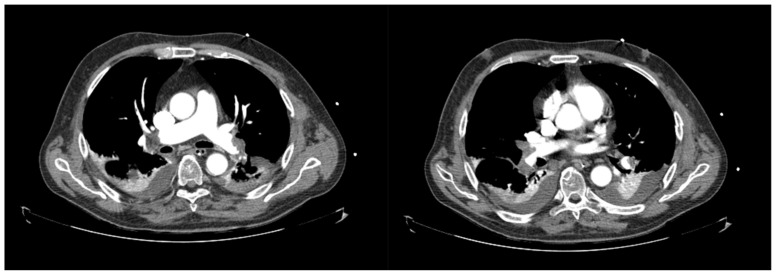
Post-procedural thoracic Angio-CT showing persisting large bilateral pulmonary embolism with significant thrombotic burden.

A subsequent computed tomography (CT) scan confirmed the described findings and reported pulmonary infarction on the right lower lobe (Figure 6).

**Figure 7:**
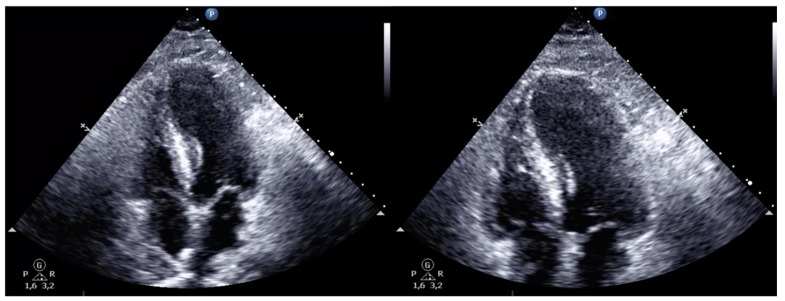
Post-procedure transthoracic echocardiogram (apical 4-chamber view) with preserved biventricular systolic function and normal-sized cardiac chambers.

The patient was admitted to the intensive care unit (ICU) under systemic anticoagulation with UFH and later switched to low-molecular-weight heparin (LMWH). Post-procedural echocardiographic reassessment showed normal-sized cardiac chambers with preserved biventricular systolic function (Figure 7, Video S10). Returned laboratory results showed an elevated high-sensitivity troponin T (313 ng/L) and Pro-BNP (6089 pg/mL). Further investigation excluded antiphospholipid syndrome and bacterial and SARS-CoV-2 infection.

Although cardiovascular recovery was immediate, neurological damage due to hypoxia was still significant. The patient had a slow but progressive neurological status improvement, however with persistent dysphagia. Respiratory function also improved rapidly, with a steep reduction in supplementary oxygen needs (at 48 h, the patient was being ventilated with 35% oxygen concentration). Also, due to recurrent nosocomial respiratory infections, extubation was difficult, and there was a need for surgical tracheostomy. Mechanical ventilatory support was suspended 12 days after the admission. The patient was later transferred to a rehabilitation unit, where he started a physical rehabilitation program. Currently, he is already able to walk with help and speak adequately.

## 3. Discussion

In this case, the patient presented with dyspnea and chest pain followed by cardiac arrest. Post-arrest ECG showed ST-segment elevation on antero-septal leads, and echocardiography showed RV dilation and dysfunction. Facing this clinical picture, STEMI suspicion was high, and the patient had unequivocal indication for emergent coronary angiography [4]. However, echocardiographic findings were discordant and raised concerns of an underlying PE, which was instrumental information for procedure conversion after the exclusion of significant epicardial CAD.

Despite being rare, it has been shown that PE can present itself with ST-segment elevation, mimicking STEMI. The most commonly affected leads are V1 to V4, as was seen in this case [6,7,14,15,16,17], but inferior lead involvement has also been reported [18]. The gold standard diagnostic test for PE is computed tomography pulmonary angiography; however, alternatively, invasive pulmonary angiography can confirm the diagnosis [3]. In the setting of ST-segment elevation, an absence of significant CAD and PE suspicion, prompt pulmonary angiography during the same procedure can be of use for quick diagnosis and therapy initiation, especially in patients in shock.

Patients presenting with high-risk acute PE, or intermediate-high risk with progressive clinical deterioration (or a lack of improvement), have an indication for reperfusion therapy. STL is usually the preferred strategy in the absence of contraindications [3,19,20]. Recently, it has been demonstrated that CDT with Penumbra^®^ Indigo^®^ (or other validated devices) has a role in the treatment and can improve the prognosis of intermediate-high-risk PE patients [11,13,21,22]. Although significant advances have been made regarding PE CDT, there is still a lack of robust evidence in the treatment of high-risk patients, especially the ones in obstructive shock. STL is still the standard of care, yet it comes at a cost of a significant risk of major bleeding and the need to consider potential contraindications [20,23]. Catheter-directed aspiration thrombectomy and low-dose local thrombolysis have been reported to be safe and effective in the treatment of hemodynamically unstable PE patients [24].

We report a case of successful reperfusion using Penumbra^®^ Indigo^®^ for aspiration thrombectomy and catheter-directed thrombolysis, with immediate hemodynamic improvement allowing withdrawal of vasopressor support and with documentation of early RV function recovery.

Despite the absence of absolute contraindications to STL in this case, we opted for CDT due to the acutely deteriorating status and the prolonged, and potentially traumatic, resuscitation efforts (relative contraindication). When a PE is diagnosed in the CCL of a center with expertise in CDT, it seems reasonable to immediately deliver therapy (mechanical and thrombolytic) in an invasive way.

Regarding the device choice, it should be stated that Penumbra^®^ Indigo^®^ System 8F was the only pulmonary thrombectomy device available at our center at the time. One could argue that, considering the high thrombotic burden, this patient could have benefited from a larger device, such as the Penumbra^®^ Lightning Indigo System (12F) or even Inari^®^ FlowTriever 2^®^. In fact, despite large bore access, the latter one showed interesting results in the FLASH registry in terms of efficacy and safety [25].

The goal of reperfusion therapy in high-risk PE is hemodynamic stabilization and improvement in gas exchange. There is often no need to extract the totality of the clot burden to accomplish it. Despite our center’s limitation at the time, we were successful in achieving this goal and in reducing mPAP and oxygen needs in a comparable way to the most recent published data [11,13,25].

## 4. Conclusions

Bedside echocardiography in patients with ST-segment elevation, post-ROSC is instrumental in raising the suspicion of acute PE. In these situations, pulmonary angiography can be considered when PE suspicion is high in a patient without significant CAD. Immediate CDT and aspiration with the Indigo^®^ system in high-risk acute PE seems safe and effective in relieving obstructive shock and restoring hemodynamics.

## Data Availability

This study generated no clinical databases. Clinical patient record data are unavailable due to privacy restrictions.

## Supplementary Materials

The following supporting information can be downloaded at https://www.mdpi.com/article/10.3390/jcdd10090374/s1, Video S1. Coronary angiography—left coronary artery (cranial view), Video S2. Coronary angiography—left coronary artery (caudal view), Video S3. Coronary angiography—right coronary artery, Video S4. Pre-procedure pulmonary angiography—right pulmonary artery, Video S5. Pre-procedure pulmonary angiography—left pulmonary artery, Video S6. Aspiration thrombectomy in the right pulmonary artery. Separator wire in the picture, Video S7. Aspiration thrombectomy in the left pulmonary artery. Separator wire in the picture, Video S8. Post-procedure pulmonary angiography—right pulmonary artery, Video S9. Post-procedure pulmonary angiography—left pulmonary artery, Video S10. Post-procedure transthoracic echocardiogram (apical 4-chamber view).
